# Novel patients with NHLRC2 variants expand the phenotypic spectrum of FINCA disease

**DOI:** 10.3389/fnins.2023.1123327

**Published:** 2023-04-27

**Authors:** Antti Tallgren, Leo Kager, Gina O’Grady, Hannu Tuominen, Jarmo Körkkö, Outi Kuismin, Martha Feucht, Callum Wilson, Jana Behunova, Eleina England, Mitja I. Kurki, Aarno Palotie, Mikko Hallman, Riitta Kaarteenaho, Franco Laccone, Kaan Boztug, Reetta Hinttala, Johanna Uusimaa

**Affiliations:** ^1^Research Unit of Clinical Medicine and Medical Research Center, Oulu University Hospital, University of Oulu, Oulu, Finland; ^2^St. Anna Children’s Hospital, Vienna, Austria; ^3^Department of Pediatrics and Adolescent Medicine, Medical University of Vienna, Vienna, Austria; ^4^St. Anna Children’s Cancer Research Institute (CCRI), Vienna, Austria; ^5^Paediatric Neuroservices, Starship Children’s Health, Te Whatu Ora Health New Zealand, Auckland, New Zealand; ^6^Department of Pathology, Oulu University Hospital, University of Oulu, Oulu, Finland; ^7^Center for Intellectual Disability Care, Oulu University Hospital, Oulu, Finland; ^8^Department of Clinical Genetics, Oulu University Hospital, Oulu, Finland; ^9^Department of Paediatrics, Center for Rare and Complex Epilepsies, Medical University of Vienna, Vienna, Austria; ^10^National Metabolic Service, Auckland City Hospital, Auckland, New Zealand; ^11^Department of Medical Genetics, Medical University of Vienna, Vienna, Austria; ^12^Mendelian Genomics, Programme in Medical and Population Genetics, Broad Institute of MIT and Harvard, Cambridge, MA, United States; ^13^Programme in Medical and Population Genetics, Broad Institute of MIT and Harvard, Cambridge, MA, United States; ^14^Analytic and Translational Genetics Unit, Massachusetts General Hospital, Boston, MA, United States; ^15^Stanley Center for Psychiatric Research, Broad Institute of MIT and Harvard, Cambridge, MA, United States; ^16^Psychiatric and Neurodevelopmental Genetics Unit, Department of Psychiatry, Massachusetts General Hospital, Boston, MA, United States; ^17^Analytic and Translational Genetics Unit, Department of Medicine, Massachusetts General Hospital, Boston, MA, United States; ^18^Department of Neurology, Massachusetts General Hospital, Boston, MA, United States; ^19^Institute for Molecular Medicine Finland (FIMM), University of Helsinki, Helsinki, Finland; ^20^Clinic for Children and Adolescents, Oulu University Hospital, Oulu, Finland; ^21^Research Unit of Internal Medicine, University of Oulu, Oulu, Finland; ^22^Center of Internal Medicine and Respiratory Medicine and Medical Research Center Oulu, Oulu University Hospital, Oulu, Finland; ^23^CeMM Research Center for Molecular Medicine of the Austrian Academy of Sciences, Vienna, Austria; ^24^Biocenter Oulu, University of Oulu, Oulu, Finland

**Keywords:** NHLRC2, whole exome sequencing, FINCA disease, neurodevelopmental disorder, macrocytic anemia

## Abstract

**Purpose:**

FINCA disease (Fibrosis, Neurodegeneration and Cerebral Angiomatosis, OMIM 618278) is an infantile-onset neurodevelopmental and multiorgan disease. Since our initial report in 2018, additional patients have been described. FINCA is the first human disease caused by recessive variants in the highly conserved *NHLRC2* gene. Our previous studies have shown that Nhlrc2*-*null mouse embryos die during gastrulation, indicating the essential role of the protein in embryonic development. Defect in NHLRC2 leads to cerebral neurodegeneration and severe pulmonary, hepatic and cardiac fibrosis. Despite having a structure suggestive of an enzymatic role and the clinical importance of NHLRC2 in multiple organs, the specific physiological role of the protein is unknown.

**Methods:**

The clinical histories of five novel FINCA patients diagnosed with whole exome sequencing were reviewed. Segregation analysis of the biallelic, potentially pathogenic *NHLRC2* variants was performed using Sanger sequencing. Studies on neuropathology and NHLRC2 expression in different brain regions were performed on autopsy samples of three previously described deceased FINCA patients.

**Results:**

One patient was homozygous for the pathogenic variant c.442G > T, while the other four were compound heterozygous for this variant and two other pathogenic *NHLRC2* gene variants. All five patients presented with multiorgan dysfunction with neurodevelopmental delay, recurrent infections and macrocytic anemia as key features. Interstitial lung disease was pronounced in infancy but often stabilized. Autopsy samples revealed widespread, albeit at a lower intensity than the control, NHLRC2 expression in the brain.

**Conclusion:**

This report expands on the characteristic clinical features of FINCA disease. Presentation is typically in infancy, and although patients can live to late adulthood, the key clinical and histopathological features are fibrosis, infection susceptibility/immunodeficiency/intellectual disability, neurodevelopmental disorder/neurodegeneration and chronic anemia/cerebral angiomatosis (hence the acronym FINCA) that enable an early diagnosis confirmed by genetic investigations.

## 1. Introduction

FINCA is an infantile-onset disease characterized by severe interstitial pulmonary fibrosis, progressive neurodegeneration, recurrent infections and chronic hemolytic anemia (Uusimaa et al., 2018). Previously, we reported two Finnish families with three affected patients with a novel early-onset multiorgan disease associated with biallelic pathogenic variants in the *NHLRC2* gene (Uusimaa et al., 2018). All had infantile-onset severe progressive disease with tissue fibrosis and progressive neurodegeneration and leptomeningeal and cerebral revascularisation on autopsy. Based on the clinical manifestations of fibrosis, neurodegeneration and cerebral angiomatosis, we proposed the acronym FINCA for the name of the disease. The defect of NHLRC2 leads to cerebral neurodegeneration and severe pulmonary, hepatic and cardiac fibrosis. However, the physiological role of the NHLRC2 protein is unknown.

Since our initial report, more FINCA patients have been diagnosed worldwide (Brodsky et al., 2020; Rapp et al., 2021; Badura-Stronka et al., 2022). Interstitial lung disease and recurrent infections are pronounced in almost all patients during infancy and can lead to death before the age of 2–3 years. Alternatively, in some cases, the progressive disease stabilizes and the neurodevelopmental disorder (NDD) evolves as the key clinical finding (Rapp et al., 2021; Badura-Stronka et al., 2022). FINCA is the first human disease known to be caused by recessive variants in the highly conserved NHLRC2. The protein has a structure suggesting an enzymatic role (Biterova et al., 2018) with three domains: an N-terminal thioredoxin-like (Trx-like) domain, followed by a six-bladed NHL repeat containing a β-propeller domain and a C-terminal β-stranded domain. The N-terminal Trx-like domain contains an unusual CCINC motif at the position of the CXXC motif, which is characteristic of oxidoreductases of the thioredoxin superfamily and commonly involved in thiol–disulfide exchange. However, no classical thioredoxin activity has been detected for NHLRC2 (Uusimaa et al., 2018; Yeung et al., 2019).

Our studies have shown that *Nhlrc2* knockout (KO) mouse embryos die during gastrulation, indicating an essential role for the protein in embryonic development (Hiltunen et al., 2022). This gastrulation or early neurulation defect is consistent with the anatomic malformations called development duplications (DD, OMIA 002103-9913)^1^ that have been observed in association with the NHLRC2 variant p.Val311Ala in Angus cattle, highlighting its function in the development of the central nervous system (Polkoff et al., 2017). Our previous findings from the compound heterozygote FINCA knockin and *Nhlrc2* KO mouse model associated hnRNP C2 and RNA metabolism with the FINCA disease pathology, suggesting that NHLRC2 plays an important role in the hippocampus (Hiltunen et al., 2020). FINCA/KO mice had increased hnRNP C2 in embryonic cortical neuronal precursor cells and in the adult hippocampus, suggesting a role for dysregulated RNA metabolism in FINCA disease.

Altered *NHLRC2* or NHLRC2 levels have been detected not only in association with FINCA disease, but also with idiopathic pulmonary fibrosis (IPF) (Boon et al., 2009; Kreus et al., 2022), lung adenocarcinoma (Ye et al., 2019), Parkinson’s disease (van Dijk et al., 2012), Alzheimer’s disease (AD) (Long et al., 2016), and sporadic amyotrophic lateral sclerosis (Andrés-Benito et al., 2020), suggesting that NHLRC2 may indeed play a role in more common pathologies. Interestingly, increased levels of NHLRC2 have been detected in the serum samples of AD patients, proposing NHLRC2 as a potential serum biomarker for the disease (Long et al., 2016).

Here, we present clinical data on five new FINCA patients and expand the phenotype of the disease. Furthermore, we present data on neuropathology and NHLRC2 expression in different brain regions in autopsy samples. We also provide pertinent details on hematological findings. Based on these data, we further delineate the clinical characteristics of FINCA disease.

## 2. Materials and methods

### 2.1. Patients and patient-derived samples

The clinical, laboratory and radiological data of five patients from three centers was collected (Clinic for Children and Adolescents, Oulu University Hospital, Oulu, Finland; St. Anna Children’s Hospital; Department of Paediatrics, Medical University of Vienna, Austria; and Starship Children’s Hospital, Auckland, New Zealand). The patients were initially identified after whole exome sequencing (WES) revealed biallelic potentially pathogenic *NHLRC2* variants and with the clinical details suggestive of FINCA disease (Uusimaa et al., 2018); the respective clinicians contacted the corresponding author (J.U.). Studies on neuropathology and NHLRC2 expression in different brain regions were performed on the available autopsy samples of three previously described deceased FINCA patients (Uusimaa et al., 2018). Written informed consent was obtained from all the parents or guardians of the patients participating in the study. The study was approved by the ethics committees of the participating centers.

### 2.2. Molecular genetic studies

Genomic DNA was extracted from peripheral blood or tissue samples of the probands, their affected siblings and available parental samples using standard methods. WES was performed at the Broad Institute of MIT and Harvard, Cambridge, MA, USA, for patient four (family three; post-mortem Sanger sequencing was performed from DNA extracted from the tissue sample of patient five), at the Department Medical Genetics, Medical University of Vienna for patient six (family four; post-mortem Sanger sequencing of DNA was performed from tissue sample for patient seven), and for patient eight (family five) as a part of the Northern Finland Intellectual Disease project led by Professor Aarno Palotie (Kurki et al., 2019). The segregation of identified NHLRC2 variants within families three and four was confirmed, whereas parental DNA was not available in family five. More detailed data are provided in the Supplementary material on the case histories.

### 2.3. Histopathological analyses of brain autopsy samples

Autopsy samples were obtained from the brains of three previously described FINCA patients (Uusimaa et al., 2018). The tissue was fixed in buffered 4% formaldehyde, routinely processed into paraffin blocks and cut into 4.0 μm sections. The autopsy samples of the patients and controls were prepared within a day after death. The control patient was 7 months old at the time of his death.

### 2.4. Histopathological analyses and immunohistochemistry of brain autopsy samples

Brain sections were stained with antibodies against NHLRC2 (Atlas antibodies, HPA038493, Bromma, Sweden). Samples were stained with a Flex-kit from Dako (Dako, Glostrup Denmark). Before application of the primary antibody, sections were heated in a microwave oven with Tris-EDTA, pH 9.0, for 15 min. After overnight incubation in +4°C with the primary antibody (1:500), a biotinylated secondary HRP Rabbit/mouse-antibody (Dako) was used. Negative control stainings were carried out by substituting non-immune rabbit or mouse primary antibody isotype control (Zymed Laboratories Inc. South San Francisco, CA, USA) and PBS for the primary antibody.

Whole-slide images were acquired with a NanoZoom S60 scanner (Hamamatsu, Hamamatsu City, Japan) in the Transgenic and Tissue Phenotyping core facility, Biocenter Oulu, University of Oulu at 40 × magnification.

## 3. Results

### 3.1. NHLRC2 variants in five novel FINCA patients

**FIGURE 1:**
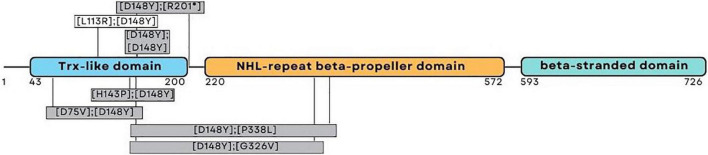
Schematic representation of the NHLRC2 domain composition and amino acid substitutions identified from the FINCA patients. The patients reported here harbored variants presented above the corresponding domain; the variants presented in gray boxes have been described previously (Uusimaa et al., 2018; Brodsky et al., 2020; Rapp et al., 2021; Badura-Stronka et al., 2022).

**FIGURE 2:**
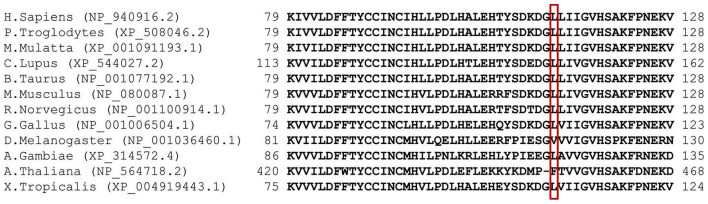
Multiple sequence misalignment of NHLRC2 spanning aa 79–128. p.L113R has been highlighted with a red box. Generated by MUSCLE (Edgar, 2004) version 3.6 (using option: -maxiters2) (Edgar, 2004).

One patient was homozygous (patient eight), while the other four were compound heterozygote for the pathogenic variant c.442G > T (NM_198514.4), leading to p.D148Y (rs201701259, NP_940916.2) in the thioredoxin-like domain of NHLRC2. Patients four and five had a frameshift variant c.601_602del (NM_198514.4), leading to p.R201fs (rs757267294, NP_940916.2), which is similar to the first FINCA patients reported by Uusimaa et al. (2018). Patients six and seven had a novel c.338T > G (NM_198514.4) variant leading to p.L113R (NP_940916.2) in the thioredoxin-like domain. The variant alters the highly conserved leucine amino acid residue and has been predicted as affecting the function of NHLRC2 (SIFT 0.0) and possibly be damaging with a PolyPhen-2 score of 0.999 (sensitivity: 0.14; specificity: 0.99) (HumDiv, PolyPhen-2 v2.2.3r406). The identified variants and their location in the protein are illustrated in Figure 1 and protein alignment in Figure 2. The genetic findings are described in Table 1.

**TABLE 1:** Genetic findings and clinical features of the five novel FINCA patients.

Patient number	4	5	6	7	8
**Study center (Country)**	**New Zealand**	**New Zealand**	**Austria**	**Austria**	**Finland**
NHLRC2 variant	p.Asp148Tyr, p.Arg201GlyfsTer6	p.Asp148Tyr, p.Arg201GlyfsTer6	p. Asp148Tyr, p. Leu113Arg	p. Asp148Tyr, p. Leu113Arg	p.Asp148Tyr, p.Asp148Tyr
Gender	Female	Female	Female	Male	Male
Ethnicity	Dutch/NZ	Dutch/NZ	Slovakian	Slovakian	Finn
Kinship	Siblings		Siblings		
Age at disease onset	1–2 months	5 weeks	2 months	At birth	At birth
Irritability	Yes	Yes	n.a.	n.a.	Yes, behavioral problems
Axial hypotonia/ muscle weakness, muscle atrophy	Yes	Yes, truncal hypotonia, generalized muscle weakness and atrophy	Yes	Yes	Yes, facial, truncal and proximal limb and shoulder muscle atrophy
Movement disorder	No	No	No	No	Yes, dystonia, ataxia, spastic diplegia
Developmental delay	Yes, since 3 years	Yes, since 6 months	Yes	Yes	Yes, since 3 years
Intellectual disability	Yes, profound intellectual disability	Yes, progressive encephalopathy	Yes	Yes	Yes, profound intellectual disability
Poor visual contact/decreased vision, strabismus	Yes	Yes	Yes	n.a.	Yes, variable eye contact, autistic features
Feeding problems	Yes	Yes	Yes, PEG since the age of 5 years	Yes	Yes, at birth
Poor weight gain, failure to thrive	Yes	Yes	Yes	Yes	No
Epileptic seizures	Yes	No	Yes	No	Yes, since 4 years
Respiratory symptoms	Yes, chronic infantile pneumonitis, tracheomalacia	Yes, chronic infantile pneumonitis, tracheomalacia	Yes, ARDS in infancy	Yes, ARDS as part of multiorgan failure	Yes, recurrent pneumonias
Recurrent infections	Yes, respiratory infections	Yes, respiratory infections	Recurrent pneumonias, but no severe pulmonary problems since infancy after initiation of antibiotic prophylaxis, transient IgG deficiency	Yes, recurrent severe infections (2x *E. coli* meningitis) at birth and in infancy	Yes, respiratory and urinary tract infections
Diarrhea/other intestinal symptoms	Yes, chronic diarrhea	Episodic diarrhea	Yes, recurrent episodes of diarrhea and vomiting	Febrile gastroenteritis before septic death	Ileal occlusion, appendicitis
Chronic anemia	Yes, several blood transfusions in infancy, CDA type II	Yes, several blood transfusions in infancy, CDA type II	Yes, hemolytic, macrocytic anemia, CDA type II like, several RBC transfusions in infancy	Yes, CDA type II like anemia, several RBC transfusions	Yes, transient macrocytic anemia
Hepatomegaly/liver dysfunction	No	Yes	No	No	No
Transient kidney dysfunction	No	Yes	No	Yes, renal failure as part of the multiorgan failure	No
Other symptoms/signs	Abnormal skin, carotenemia appearance	No	Progressive microcephaly (OFC 35 cm/+1 SD at birth, and 43 cm/-3.5 SD at the age of 2.5 years)	Plexus paresis, Horner syndrome	Pectus excavatum, neuromuscular scoliosis, recurrent accidents
Overall progressive disease course	Relatively stable, pulmonary problems improved and no severe infections since 2 years	Yes, progressive respiratory and multiorgan failure	Relatively stable, drug-resistant seizures	Yes, progressive respiratory and multiorgan failure	Yes, slight neurological progression
Current age/age at death	Alive 19 years	Deceased 11 months	Alive 6 years	Deceased 10 months	Alive 61 years

NZ, New Zeland; n.a., not available; PEG, percutaneous endoscopic gastrostomy; ARDS, acute respiratory distress syndrome; CDA, congenital dyserythropoietic anemia; RBC, red blood cell; OFC, occipitofrontal head circumference; SD, standard deviation.

### 3.2. Clinical, laboratory and radiological findings of the five novel FINCA patients

We identified five novel FINCA patients [these patients are numbered here chronologically as patients 4–8 based on our initial report on the first three FINCA patients (patients 1–3) described in Uusimaa et al. (2018)]. The clinical features of the new FINCA patients are summarized in Table 1, with laboratory and radiological findings in Table 2. All five patients presented at birth or during the first months (range from birth to 2 months) and their clinical phenotypes resembled the phenotypes of the first previously published FINCA patients (Uusimaa et al., 2018). The clinical features seen in all the patients included axial hypotonia, developmental delay, visual problems (including poor visual contact, decreased vision or strabismus), feeding problems, recurrent infections, respiratory symptoms, diarrhea/other intestinal symptoms and macrocytic anemia. The majority of the patients manifested poor weight gain and failure to thrive (4/5, 80%), epileptic seizures (3/5, 60%), and irritability (3/5, 60%), while transient liver dysfunction, transient kidney dysfunction, plexus paresis, Horner syndrome and abnormal carotenemia skin color were seen in individual patients. The eldest patient (currently 61 years old, patient eight) also has progressive muscular atrophy leading to neuromuscular scoliosis, pectus excavatum, ataxia and spastic diplegia. He had drug-resistant epileptic seizures and behavioral problems, including aggressive outbursts. Two patients with compound heterozygous NHLRC2 variants (patients five and seven) died because of progressive respiratory and multiorgan failure at the age of 10–11 months, whereas their siblings were alive, with current ages of 6 (patient six) and 19 years (patient four) and were relatively stable with fewer respiratory problems and infections since the age of 2.

**TABLE 2:** Laboratory and imaging findings of five FINCA patients with the *NHLRC2* variants.

Patient number	4	5	6	7	8
**Study center (Country)**	**New Zealand**	**New Zealand**	**Austria**	**Austria**	**Finland**
**Blood analyses**					
B-hemoglobin	88–130 g/l (ref. 117–155 g/l)	65–111 g/l (ref. 90–130 g/l)	65–109 g/dl (ref. 100–143 g/l)	88–130 g/l (ref. 100–155 g/l)	120–130 g/l (ref. 134–167 g/l)
E-MCV	95–105 fl (ref. 82–98 fl)	105–115 fl (rf. 71–87 fl)	85–108 fl (ref. 75–85 fl)	85–91 fl (ref 74–106 fl)	101 fl (ref. 82–98 fl)
E-MCH	24–38 pg (ref. 27–33 pg)	28–40 pg (ref. 23–31 pg)	29–37 pg (ref. 25–35 pg)	29–32 pg (ref. 27–34 pg)	35 pg (ref. 27–33 pg)
E-MCHC	–	–	33–38 g/dl (ref. 30–37 g/dl)	33–36 g/dl (ref. 28–31 g/dl)	341 g/l (ref. 320–355 g/l)
**Immunoglobulins**					
IgG	n.a.	3.5 g/l (ref. 3–10 g/l)	2.29 g/l (ref. 2.44–11.8 g/l)	4.92 g/l (ref. 2.9–7.7 g/l)	n.a.
IgM	n.a.	0.05 g/l (ref. 0.1–1.2 g/l)	0.14 g/l (ref. 0.4–1.8 g/l)	0.35 g/l (ref. 0.11–0.76 g/l)	n.a.
IgA	n.a.	0.92 g/l	0.5 g/l (ref. 0.48–3.45 g/l)	0.37 g/l (ref. 0.33–1.25 g/l)	n.a.
**Imaging**					
X-rays/CT	CT chest at 17 years: a number of pneumatoceles especially in right lower lobe	Chest X-rays: widespread variable infections/ pneumonitis	ARDS at 4 months	n.a.	–
Brain MRI	At 17 years: severe generalized atrophy with fronto-temporal prominence, but affecting also parietal and occipital lobes and cerebellum; the corpus callosum is severely atrophied	At 11 months: generalized atrophy with mild hyperintensity of the parieto-occipital white matter	At 2 years: arachnoidal cyst in the left temporal region and atrophy of the left temporal lobe	At 2 months: hydrocephalus, mesiotemporal atrophy	–
Tissues biopsy analyses	n.a.	Bone marrow aspirate: markedly increased erythropoiesis, marked binuclearity of erythroblasts. Marked increase in eosinophils >15%-suggestive of CDA type II Muscle biopsy: normal–mild type II atrophy. Normal EM Liver biopsy: normal apart from some vacuolated cells, no fibrosis, cirrhosis Lung biopsy: severe distortion lung architecture, severe septal thickening, foamy macrophages, markedly hyperplastic type II pneumocytes (dx chronic pneumonitis of infancy) Normal ciliary biopsy	Bone marrow aspirate at age of 6 weeks: dyserythropoiesis, marked binuclearity (>15%) and a few multinuclear erythroblasts; suggestive of CDA type II	n.a.	–

Ref, reference limits; n.a., not available; CT, computed tomography scan; ARDS, acute respiratory distress syndrome; CDA, congenital dyserythropoietic anemia; EM, electron microscopy; dx, dexter.

**FIGURE 3:**
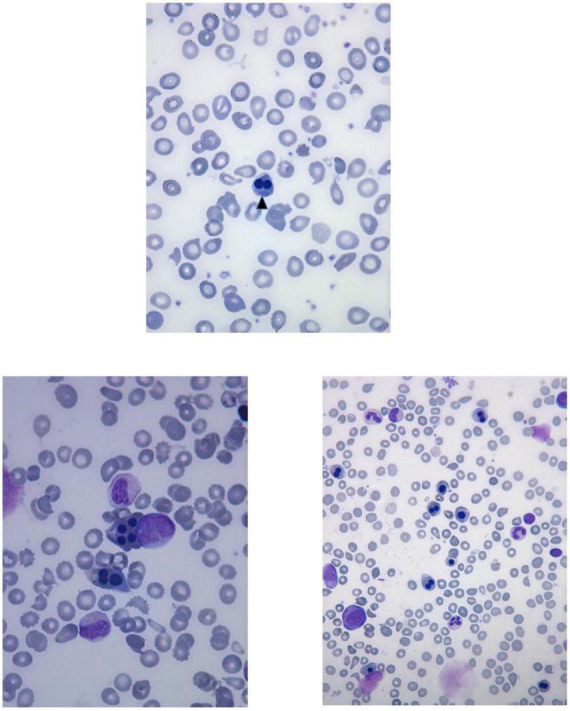
Peripheral blood smear of the patient six shows anisocytosis, poikilocytosis, fragmentocytes and binucleated normoblast (arrowhead). Bone marrow aspirates of the patient six show multinucleated erythroid precursors and dyserythropoiesis.

Laboratory investigations (Table 2) revealed macrocytic anemia in all five patients, with the bone marrow aspirates in two patients (patients five and six, Table 2) showing increased erythropoiesis or dyserythropoiesis, marked bi- and multinuclearity of erythroblasts and increased eosinophils suggestive of congenital dyserythropoietic anemia (CDA type II) (Figures 3A, B). Four out of five patients needed several red blood cell transfusions in infancy. Furthermore, immunodeficiency with decreased immunoglobulin levels was detected in two patients.

**FIGURE 4:**
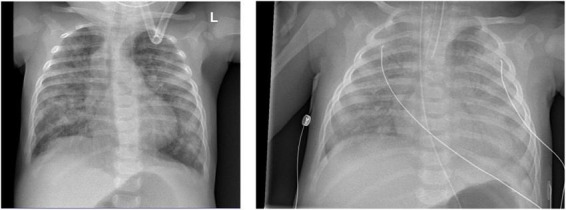
Chest X-ray of patient six at 4 months of age shows lung infiltrations and development of acute respiratory distress syndrome (ARDS, pneumocystis carinii and streptococcus pneumonia).

**FIGURE 5:**
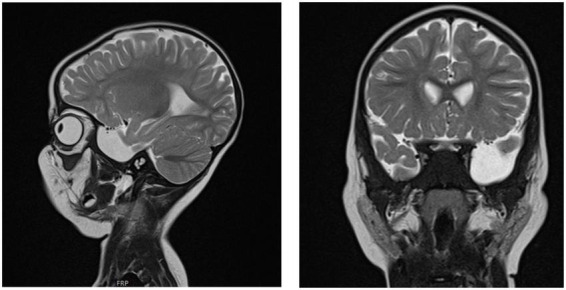
Brain MRI of patient six at the age of 2 years. T2 sagittal and coronal MRIs show an arachnoidal cyst in the left temporal region and atrophy of the left temporal lobe.

**FIGURE 6:**
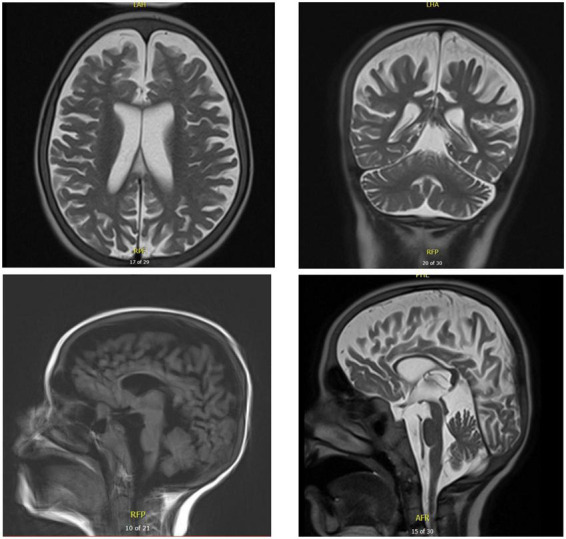
Brain MRI of patient five at the age of 17 years. T2 axial and coronal MRIs show general atrophy with normal white matter signal. Sagittal T1 and T2 brain MRIs show supra- and infratentorial cerebral atrophy, with marked thinning of the corpus callosum.

Chest X-ray (Figure 4) and chest computed tomography demonstrated acute respiratory distress syndrome (ARDS) in patient six (at 4 months of age), widespread variable infections/pneumonitis in patient five and several pneumatoceles in patient four at the age of 17 years. Lung biopsy of patient six during infancy showed severely distorted lung architecture with severe septal thickening, foamy macrophages and markedly hyperplastic type II pneumocytes (chronic pneumonitis of infancy). Brain magnetic resonance imaging (MRI) revealed an arachnoid cyst in the left temporal region and atrophy of the left temporal lobe in patient six at 2 years of age (Figure 5), hydrocephalus and mesiotemporal atrophy in patient seven at 2 months of age and severe generalized atrophy with fronto-temporal prominence, but also affecting the parietal and occipital lobes and cerebellum, and severely atrophied corpus callosum in patient four at the age of 17 years (Figure 6).

### 3.3. Histopathological findings in the brains of FINCA patients and NHLRC2 expression in humans

Immunohistochemical NHLRC2 expression was detected broadly in all the studied cells, excluding endothelial cells, where the expression was not seen in any tissues (Table 3). Expression was cytoplasmic in the neuronal, glial and meningothelial cells and in cells of the choroid plexus. In ependymal cells, the expression was apical. The strongest intensity of staining was found in neurons. In glial cells, the intensity was lower, but in both types of cells, the expression was widespread in different regions of the brains. Supplementary Figure 1 demonstrates the NHLRC2 expression in the patients’ brain autopsy samples. The intensity of the expression varied between the patients and between the patients and the control. Overall expression was wider and slightly stronger in the tissues of the control, but interestingly, it was the opposite in Purkinje cells, in which expression tended to be lower (Table 3). Furthermore, stronger expression in neuropil was occasionally observed in the control’s tissues, particularly in the temporal and parietal lobes of the brain. A comparison of NHLRC2 expression in patient and control samples is demonstrated in Supplementary Figures 2, 3.

**TABLE 3:** Expression of the NHLRC2 in different brain regions and cell types in the deceased FINCA patients and in the control samples.

	Neu	Gli	Men	End	Epe	Pil	Cho	Neu	Gli	Men	End	Epe	Pil	Cho	Neu	Gli	Men	End	Epe	Pil	Cho	Neu	Gli	Men	End	Epe	Pil	Cho
A					-		-					-		-					-		-					-	-	-
B					-		-					-		-					-		-					-		-
C					-		-					-		-	x	x	x	x	x	x	x	x	x	x	x	x	x	x
D					-		-							-	x	x	x	x	x	x	x					-		-
E					-		-					-		-	x	x	x	x	x	x	x	x	x	x	x	x	x	x
F					-		-					-		-					-		-					-		-
G																												-
H			-											-	x	x	x	x	x	x	x	x	x	x	x	x	x	x
I														-	x	x	x	x	x	x	x			-				-
J			-											-	x	x	x	x	x	x	x	x	x	x	x	x	x	x
K														-							-							-
L			-											-							-							-
M			-																									
N														-														
S	x	x	x	x	x	x	x							-							-							-
	Patient 1							Patient 2							Patient 3							Control						

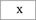
 No tissue available.

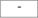
 No representative cells.

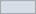
 No expression.

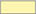
 Low expression.

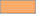
 Medium expression.

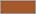
 Strong expression.

A, frontal lobe; B, temporal lobe; C, cingulate gyrus; D, parietal lobe; E, pre- and postcentral gyrus; F, occipital lobe; G, hippocampus; H, hypothalamus; I, basal ganglia; J, thalamus; K, middle brain; L, pons; M, medulla oblongata; N, cerebellum; S, spinal cord; Neu, neurons; Gli, glial cells; Men, meningothelial cells; End, endothelial cells; Epe, ependymal cells; Pil, neuropil; Cho, choroid plexus.

## 4. Discussion

We have reported on five novel patients from three unrelated families with FINCA disease. Furthermore, we present further data on neuropathology and, for the first time, NHLRC2 expression in different human brain regions in the autopsy samples of one control and the deceased FINCA patients originally described by Uusimaa et al. (2018).

### 4.1. Clinical phenotypes of novel FINCA patients compared with previously published cases

Based on the literature and novel FINCA patients described in the present study, all the patients with the *NHLRC2* gene variants presented with developmental delay, along with variable axial hypotonia, muscle weakness, progressive muscular atrophy (leading to scoliosis), eating problems, poor eye contact, strabismus, seizures, behavioral problems and various types of movement disorders (ataxia, dystonia, cerebgral palsy, tremor and stereotypic hand movements). Thus, NHLRC2 is clearly essential for normal human nervous system function.

The present study, together with the so far reported FINCA cases, underlines how chronic macrocytic anemia, immunodeficiency, recurrent infections and pneumonias causing respiratory distress are the key clinical features of FINCA disease. Notably, recent work has identified NHLRC2 as a potent regulator of phagocytosis and filopodia formation (Haney et al., 2018). Furthermore, NHLRC2-deficient macrophages have been shown to undergo morphological changes and are resistant to certain bacteria *in vitro* (Yeung et al., 2019). Our patients suffered from chronic, partly lethal infections, but if this is related to the above-identified defect in macrophages, it has to be investigated further. In addition, NHLRC2 associates with p190RhoGAP and alters the activation levels of the cytoskeleton regulator RhoA (Haney et al., 2018). Erythroid-specific deletion of RhoA in mice was embryonic lethal because of severe anemia, and the primitive red blood cells were macrocytic, poikilocytes and frequently multinucleated (Konstantinidis et al., 2015). Binucleated and multinucleated erythroid cells are the key features in certain types of CDAs (King et al., 2022). Interestingly, we observed an erythroid lineage phenotype similar to congenital dyserythropoietic anemia type II (CDA II) in the two patients who had undergone bone marrow examinations (patients five and six) (Figure 3). Moreover, there was marked anisocytosis and poikilocytosis in the peripheral blood smear observed in patient six. It is also notable that four out of five patients required RBC transfusions because of severe anemia during infancy. Clearly, further investigations are necessary to elucidate the role of NHLRC2 in RBC development and membrane stability. In patient six, the initial lead-pathologies were severe anemia associated with feeding problems, failure to thrive and muscular hypotonia; hence, hematologists should keep ultra-orphan diseases like CDAs, FINCA syndrome and CAD-associated uridine responsive epileptic encephalopathy in the differential diagnostic repertoire.

Combining the clinical features of our five novel patients with the previously described 13 patients (Uusimaa et al., 2018; Brodsky et al., 2020; Rapp et al., 2021; Badura-Stronka et al., 2022) reveals the following: 10 patients have been reported as being alive (age between 4 and 61 years at the time of the publication), while eight were deceased (age at death between 10 months and 2 years, 5 months). Eight of the 18 patients were male. All presented with axial hypotonia and developmental delay, usually severe. Most had recurrent infections, including recurrent pneumonias. Common presenting features included irritability (including impulsive behavior and aggressivity in older patients), movement disorders (dystonia/ataxia/tremor or stereotypic movements), failure to thrive, visual problems, feeding problems, episodic diarrhea or other intestinal problems, macrocytic anemia and an increased risk for respiratory distress and respiratory support during pneumonia. Epileptic seizures and immunodeficiency were also common features. Epilepsy phenotypes, electroencephalography (EEG) characteristics and brain MRI findings of FINCA patients have been presented in Supplementary Table 1. Furthermore, some patients exhibited transient liver dysfunction, hepatomegaly, transient kidney dysfunction and cardiac manifestations. Other features included hypothyroidism in two patients, pectus excavatum in two patients, tracheomalacia in one patient, cholelithiasis in one patient and an abnormal color of the skin in one patient. Chest radiography showed ARDS and chronic pneumonitis, with brain MRI revealing atrophy of the corpus callosum. A severe general cerebral and cerebellar atrophy in a patient at the age of 17 years suggested progressive neurodegeneration.

In previous publications, four patients were identified with homozygosity for the p.D148Y variant, as in our patient with the current age of 61 years (patient eight). All five patients were alive, and the ages of four previously reported cases varied between 7 and 14 years at the time of the publication (Rapp et al., 2021; Badura-Stronka et al., 2022). Thus, the phenotype could be differentially affected, depending on the loci of the rare damaging variants within the *NHLRC2* gene. On the other hand, there was significant heterogeneity in the clinical presentation and outcome, even between the siblings with the same variants. Therefore, we conclude that the severity of disease seems to be modified by additional genetic and environmental factors, including exposure to infections and those related to developmental biology that need to be studied further.

### 4.2. Neuropathology of FINCA patients and NHLRC2 expression in the human brain

Autopsy samples of three previously described FINCA patients showed brain atrophy, white matter neuronal degeneration and angiomatosis such as vascularization and congestion (Uusimaa et al., 2018). The NHLRC2 expression data revealed that the control samples showed overall stronger NHLRC2 expression in neuronal and glial cells than in the patients (Table 3). On the other hand, we identified a clearly higher expression of NHLRC2 in the Purkinje cells of FINCA patients compared with the controls (Supplementary Figure 2). This finding is very interesting because Purkinje cells play a central role in cerebellar development and all cerebellar circuits. Because FINCA disease is a neuroimmunological disease, one hypothesis could be based on the link between progressive neurodegeneration and impaired peripheral immune responses, as exemplified by a recent study demonstrating that the selective loss of Purkinje cells induces specific peripheral immune alterations by attracting leukocytes toward and into the cerebellum of a Purkinje cell degeneration mouse model (del Pilar et al., 2021). In their study, del Pilar et al. (2021) also suggested that this phenomenon could serve as an early biomarker of cerebellar degeneration and be responsible for an increased susceptibility to infections. Furthermore, the authors referred to several previous studies that have shown that the progression of neurodegenerative diseases has been reciprocally associated with impairments in peripheral immune responses and responsible for an increased susceptibility to infections, as exemplified by multiple sclerosis, AD, Huntington’s disease, Parkinson’s disease and amyotrophic lateral sclerosis (Scherzer et al., 2007; Björkqvist et al., 2008; Ciaramella et al., 2016; Fakhoury, 2016; Liu and Wang, 2017), and the identification of increased amounts of proinflammatory cytokines in cerebrospinal fluid and blood of patients with Alzheimer’s and Parkinson’s (Boyko et al., 2017; Chen et al., 2018).

The role of NHLRC2 in the function of the normal central nervous system and other organs remains unclear, but it is known to be involved in cytoskeletal organization and vesicle transport and has an important role in the maintenance of multiorgan homeostasis (Paakkola et al., 2018; Uusimaa et al., 2018). Additionally, NHLRC2 dysfunction contributes to the evolving neurodegeneration observed in the previously published FINCA patients and was clearly demonstrated in the current study. Our findings indicate that the pathological variants of *NHLRC2* influence protein expression in various regions of the human brain, hence causing variable neurological symptoms.

### 4.3. NHLRC2 variants associated with FINCA disease and disease models

In previous studies on NHLRC2, the thioredoxin-like domain was found to interact with a proenzyme form of caspase-8, and caspase-8 cleaves NHLRC2 *in vitro*. Excess reactive oxygen species (ROS) production led to a caspase-8-mediated decrease in NHLRC2 protein levels, leading to apoptotic cell death in colon cancer cells, suggesting an important role for NHLRC2 in the regulation of ROS-induced apoptosis (Nishi et al., 2017). NHLRC2 has been indicated as being a key regulator in phagocytosis and is suggested to act through controlling actin polymerization, cytoskeletal organization and filopodium formation (Haney et al., 2018; Paakkola et al., 2018; Yeung et al., 2019). Several genes involved in actin cytoskeletal arrangements were downregulated in the NHLRC2 mutant macrophages, and a co- immunoprecipitation experiment confirmed the interaction of NHLRC2 with FRY-like transcription coactivator (FRYL), which is known to play a role in actin cytoskeleton regulation (Yeung et al., 2019).

A knockin mouse model for FINCA with pathogenic missense variant—c. G442T; p.D148Y—was generated in our research group by using the CRISPR/Cas9 technique (Hiltunen et al., 2020). This mouse line—C57BL/6N*Nhlrc2*^em1Rthl^—has been crossed with the *Nhlrc2* KO mouse line to mimic the compound heterozygous genotype of the FINCA patients. *in situ* hybridization (ISH) of *Nhlrc2* showed ubiquitous expression throughout the adult brain of 32-week-old male mice, with the most prominent expression in cerebellar granule cells, followed by granule cells in the dentate gyrus and then by pyramidal cells in the hippocampal CA1 layer and layer 2 of the piriform cortex. ISH of FINCA/KO mice brain revealed a similar expression pattern of the mutated *Nhlrc2* mRNA to that of the wild-type (Hiltunen et al., 2020).

## 5. Conclusion

We report five new patients with FINCA disease, demonstrating strikingly similar key phenotypic features with the previously published FINCA patients (Brodsky et al., 2020; Rapp et al., 2021; Badura-Stronka et al., 2022). Furthermore, our data underline the importance of performing hematological and immunological studies on all patients with biallelic pathogenic NHLRC2 variants. Immunoglobulin treatment and additional vaccinations (e.g., pneumococcal vaccine and varicella immunoglobulin) and antibiotic prophylaxis (for pneumocystis carinii, etc.) are recommended if immunodeficiency is detected. Our results further demonstrate that biallelic *NHLRC2* gene variants cause infantile-onset FINCA disease, facilitate the early diagnosis and eventually provide clues how to improve the management of this severe multiorgan disease. Based on the current knowledge on the phenotypic spectrum of FINCA disease, we recommend considering WES analysis for patients with a history of infantile-onset cerebropulmonary manifestations as well as infantile-onset epileptic encephalopathies without pulmonary manifestations.

The clinical and histopathological characteristics of NHLRC2-related diseases, namely fibrosis, infection susceptibility/immunodeficiency/intellectual disability, neuro-developmental disorder/neurodegeneration, and chronic anemia/cerebral angiomatosis can be summarized by the acronym FINCA.

## Data availability statement

The original contributions presented in this study are included in the article/Supplementary material, further inquiries can be directed to the corresponding author.

## Ethics statement

The studies involving human participants were reviewed and approved by the Ethics Committee of the Northern Ostrobothnia Hospital District, Oulu, Finland. Written informed consent to participate in this study was provided by the participants’ legal guardian/next of kin.

## Author contributions

AT, HT, OK, JK, RK, RH, and JU contributed to the conception and design of the study. AT, LK, GO’G, JK, OK, MF, CW, JB, FL, KB, and JU collected and interpreted clinical, neuroradiological, and laboratory data on patients. AT and HT performed neuropathological analyses. OK, EE, JB, FL, MK, and AP organized and performed molecular genetic analyses. AT, RH, and JU wrote the first draft of the manuscript. LK, GO’G, HT, and MH wrote sections of the manuscript. All authors contributed to manuscript revision, read, and approved the submitted version.

## Footnotes

1
https://omia.org/OMIA002103/9913/

## Supplementary material

The Supplementary material includes case histories of five novel FINCA patients, three figures on neuropathological findings in autopsy samples of the three previously published FINCA patients and a table presenting epilepsy phenotypes, EEG characteristics and brain MRI findings in novel and previously published FINCA patients.

Supplementary Figure 1 Immunohistochemical NHLRC2 expression in brain autopsy samples of patients. **(A)** Strong expression in thalamic neurons. **(B)** Medium strength and widespread expression in the glial cells of the middle brain. Some glial cells are weakly positive or negative for NHLRC2. **(C)** Cerebellar overview and higher magnifications of the cell layers and Purkinje cells. Strong expression in Purkinje cells. **(D)** Meningothelial cells are weakly positive for NHLRC2. **(E)** Neurons of the inferior olivary nucleus have medium to strong expression. **(F)** Ependymal cells had apical expression and were weakly positive or negative for NHLRC2. **(G)** Dentate gyrus of the hippocampus and mild expression in neurons. **(H)** Expression in choroid plexus cells and periventricular neurons.

Supplementary Figure 2 NHLRC2 expression was compared in different brain regions between patients and control. **(Frontal lobe)**: Medium expression in neurons. There was no significant difference between the patients and control. **(Temporal lobe)**: The intensity of the staining is stronger in the control compared with the patients. Medium to strong expression in the neuropils of the control. **(Pons)**: Medium to strong expression in the neurons. The control group had slightly stronger expression compared with the patients. **(Cerebellum)**: Strong expression in the Purkinje cells of the patients and mild in the cells of the control. **(Medulla oblongata)**: Stronger expression in the neurons of the control group compared with the patients. **(Basal ganglia)**: The expression and intensity of the staining is stronger in the control compared with the patients.

Supplementary Figure 3 NHLRC2 expression was compared in different brain regions between the patients and control. **(Frontal lobe)**: Mild-to-medium expression in the glial cells of the patients and medium expression in the glial cells of the control. **(Occipital lobe)**: Mild to strong expression in the glial cells. Slightly stronger expression in the control group compared with the patients. **(Parietal lobe)**: Stronger expression and more frequent occurrence of glial cells in the control. **(Spinal cord)**: Strong expression in neurons in both the patients and control. **(Ependymal cells)**: Negative to mild apical expression in the ependymal cells of the patients and medium apical expression in the ependymal cells of the control.
