# Supplementary material for: Novel patients with NHLRC2 variants expand the phenotypic spectrum of FINCA disease
Source: Front Neurosci. 2023 Apr 27;17:1123327. doi: 10.3389/fnins.2023.1123327 (PMC10173879; doi:10.3389/fnins.2023.1123327)
Supplement: Supplementary file 1 [file Table_1.docx]

Supplementary Table 1: Epilepsy phenotypes, EEG characteristics and brain MRI findings in FINCA patients.

|  | **Patient**  **number** | **Nationality/**  **sex/**  **age at death** | **Epileptic seizures/**  **age at seizures onset** | **Type of seizure** | **EEG** | **Response to antiseizure medication** | **Brain MRI** | **Head circumference /microcephaly** |
| --- | --- | --- | --- | --- | --- | --- | --- | --- |
| Uusimaa et al. 2018 (described as patients 1-3) | **1** | Finnish/M/  deceased 1yr 9mo | yes/7 mo | at 7 mo: atypical absences | slow background activity and frontal bilateral rhythmic high-amplitude sharp delta transients, indicating metabolic encephalopathy | n.a. | 7 mo: a thin corpus callosum | decrease in head circumference from -0.5 SD to -1.2 SD |
|  | **2** | Finnish/M/  deceased 1yr 1mo | no seizures | | n.a. | n.a. | 10 mo: a thin corpus callosum and slightly dilated lateral ventricles and cortical sulci | n.a. |
|  | **3** | Finnish/M/  deceased 1yr 2mo | yes/4 mo | at 4 mo: suspected seizure during an influenza B infection | normal during wakefulness, but unusually monotonic during sleep, during which it consisted of 4-Hz delta waves without normal sleep spindles and vertex waves | n.a. | 10 mo: a thin corpus callosum, slightly enlarged lateral ventricles and cortical sulci, as well as T2-weighted axial MRI revealed a mildly increased signal intensity of the globus pallidus | n.a. |
| Current study | **4** | Dutch, NZ/F/  alive | yes/3 yr | n.a. | poorly formed background rhythm with excessive slow activity and the presence of multifocal independent epileptiform discharges | n.a. | at 17 yr: a thin corpus callosum, severe generalised atrophy with fronto-temporal prominence, but affecting also parietal and occipital lobes and cerebellum | n.a. |
|  | **5** | Dutch, NZ/F/  deceased 11mo | no seizures | | n.a. | n.a. | at 11 mo: generalised atrophy with mild hyperintensity of the parieto-occipital white matter | n.a. |
|  | **6** | Slovakian/F/  alive | yes/3 yr 10mo | tonic-myoclonic | multifocal and generalized, corresponding with the LGS | despite broad anticonvulsive medication, the patient has three to five tonic-clonic seizures per day that persist spontaneously | at 2 yr: arachnoidal cyst in the left temporal region and atrophy of the left temporal lobe | microcephaly (OFC35 cm/ + 1 SD at birth, and 43 cm/-3.5 SD at the age of 2,5 yr) |
|  | **7** | Slovakian/M/  deceased 10mo | no seizures | | n.a. | n.a. | at 2 mo: hydrocephalus, mesiotemporal atrophy | n.a. |
|  | **8** | Finnish/M/  alive | yes/4 yr | Focal onset GTC, atypical absences, myoclonia, atonic seizures and aggressive bursts | multifocal epileptic activity with bifrontal predominance, generalized slowing with bursts of spike and wave discharges and bursts of generalized paroxysmal fast activity corresponding to LGS | no GTCs during CBZ treatment, but no response to aggressive bursts. Atypical absences from 52 years followed by reoccurrence of GTCs seizures, and CBZ was replaced by LTG. A good response to GTCs with BNZ without progression to SE | n.a. | n.a. |
| Brodsky et al. 2020 | **9** | Ukrainian/M/  deceased 2yr 5mo | yes/1yr 9mo | n.a. | epileptogenic myoclonus | LEV, no additional information | a thin corpus callosum, fronto-temporal atrophy, dilated lateral ventricles and cortical sulci | n.a. |
| Rapp et al. 2021 (described as propands 1-6) | **10** | Greek/M/  deceased 1yr 10mo | yes/9 mo | n.a. | n.a. | n.a. | brain atrophy with subsequent hydrocephaly with increased subdural spaces | n.a. |
|  | **11** | Greek/F/  deceased 1yr 5mo | yes/n.a. | seizure during intubation in hospital | n.a. | n.a. | n.a. | n.a. |
|  | **12** | Greek/F/  alive | yes/n.a. | seizure during intubation in hospital | n.a. | n.a. | normal | n.a. |
|  | **13** | Belgian/F/  alive | no seizures | | n.a. | n.a. | a thin corpus callosum and generalized cortical and subcortical atrophy |  |
|  | **14** | Jordanian/M/  alive | no seizures | | n.a. | n.a. | n.a. | n.a. |
|  | **15** | Jordanian/F/  alive | no seizures | | n.a. | n.a. | n.a. | n.a. |
| Badura-Stronka et al. 2022 (described as P11-P13) | **16** | Polish/F/  alive | no seizures | | in right posterior temporo‐occipital regions during wakefulness, a disturbed spatial organization, high‐voltage, with high superimposed fast activity at 14–18 Hz, up to 150 uV; single and groups of sharp waves up to 280 uV leads. During sleep, series of irregular sharp‐and‐slow‐wave and spike–wave complexes at 3.5–4 Hz, up to 615 uV | n.a. | normal | n.a. |
|  | **17** | Polish/F/  alive | yes/4 yr 6mo | focal seizures with secondary generalization (after awakening, the first episode starts with gagging, eye deviation, after 8 minutes atonia, cyanosis, loss of consciousness); similar episodes1–2 times a year” | single and groups of sharp‐and‐slow‐wave complexes, up to 700 uV in the posterior area with a predominance of the right side, During sleep numerous generalized series of slow waves at 2–2.5 Hz and spike–wave complexes, up to 450 uV, with a predominance in the central areas | worsening of behavior after initiation of LEV, TPM and LTG (therefore withdrawn by the parents) | venous anomaly in the left cerebellar hemisphere | n.a. |
|  | **18** | Polish/F/  alive | yes/4 yr | focal seizures with secondary generalization, SE three times | irregular alpha waves at 8–10 Hz, up to 180 uV in the posterior leads during wakefulness, with a predominance of fast beta activity in all leads. In posterior occipital‐parietal–temporal leads, discharges of high‐voltage sharp waves, spikes, and polyspikes, sometimes within a slow wave complex, up to 400–500 uV. During sleep, multiple generalized discharges in the form of sharp waves, polyspikes, and spike‐and‐slow‐wave complexes | good response to VPA, no response to LEV (withdrawn) | a thin corpus callosum, thickening of bone marrow in skull bones | n.a. |

Abbreviations: M, male; F, female; yr, years; mo, months; n.a., not available; EEG, electroencephalography; MRI, magnetic resonance imaging; LGS, Lennox-Gastaut syndrome; GTC, generalized tonic-clonic; CBZ, carbamazepine; LEV, levetiracetam; TPM, topiramate; LTG, lamotrigine; VPA, valproic acid; BNZ, benzodiazepines; OFC, occipitofrontal head circumference; SD, standard deviation.
