# Supplementary material for: Novel patients with NHLRC2 variants expand the phenotypic spectrum of FINCA disease
Source: Front Neurosci. 2023 Apr 27;17:1123327. doi: 10.3389/fnins.2023.1123327 (PMC10173879; doi:10.3389/fnins.2023.1123327)
Supplement: Supplementary file 2 [file Data_Sheet_1.docx]

+Supplementary Material, Case reports of 5 novel FINCA patients

# Case report

***Patient 4 (Family 3)*** was born 3 years after her sister. In infancy, she had similar, albeit less severe, problems with a CDA type II blood picture, hence requiring intermittent transfusions and having failure to thrive, chronic diarrhoea, tracheomalacia and a chronic pneumonitis of infancy. She had a convergent squint and a moderate developmental delay. She never became severely unwell; however, her pulmonary problems improved, and from two years of age, she has not been acutely significantly unwell.

At the age of 3 years, severe developmental delay was noticed. She could sit unsupported and stand if held. She had abnormal jerking movements. At the age of 6 years, she remained generally well from an infectious and pulmonary point of view. She had a stable low-normal haemoglobin, although the blood film remained very abnormal with widespread polychromatic, oval, teardrop and dysplastic cells with cell fragments. She had a profound developmental delay, and severe failure to thrive Her skin had an unusual carotenemic appearance.

***Laboratory investigations and imaging.*** She had increased hepatocyte glycogen on her liver biopsy. EEG at the age of 3 years showed a poorly formed background rhythm with excessive slow activity and the presence of multifocal independent epileptiform discharges. WES analysis revealed compound heterozygous variants p.Asp148Tyr and a frameshift 2-bp deletion p.Arg201GlyfsTer6 in the *NHLRC2* gene. Segregation confirmed that one variant was inherited from each parent, both of whom have distal European ancestry (Dutch, Scottish/English) but no known Finnish connection.

***Current status.*** Currently at the age of 19 years, she remains generally well and lives at home with her parents. She has made little developmental progress and is noncommunicative but is aware of her surroundings and enjoys music and personal interaction. She has short stature and feeding difficulties but is relatively stable. She is spoon-fed pureed food, with her weight and height stable below the 1st centile. She has infrequent lower respiratory problems and a right lower lobe-septated pneumatocele. She continues to have an unexplained light yellow/orange skin tone.

# Case report

***Patient 5* *(Family 3)*** was born at term to nonrelated English–Dutch parents. She weighed 2860 grams. In the neonatal period, she was noted as having significant anaemia, and over the following 2 months, she required three blood transfusions. She proceeded to have frequent hospital admissions with lower respiratory tract infections. This was associated with poor feeding, diarrhoea and failure to thrive.

At 6 months of age, there were concerns regarding her development. She had truncal hypotonia and appeared mildly weak. She had good early babble and was reaching out and rolling over. At 11 months of age, she was admitted to paediatric intensive care with severe lower respiratory tract symptoms. She had marked hypotonia, generalised muscle wasting and tracheomalacia. She became ventilator dependent and encephalopathic. She failed to respond to intravenous antibiotics, antivirals and steroids and died of respiratory and multiorgan failure at 11 months of age. She had dyserythropoietic anaemia.

***Laboratory investigations and imaging***. Her blood film during the neonatal period showed a mixed picture of microcytes, macrocytes and irregularly shaped polychromatic cells. A bone marrow aspirate revealed marked erythroid hyperplasia, with a striking binuclearity present primarily in the late erythroblasts. The features were thought to be consistent with congenital dyserythopoietic anaemia (CDA) type II. A wide range of biochemical, haematological, metabolic, cytogenetic and immunological investigations failed to find the underlying cause for her symptoms. Chest X-ray and CT at 11 months showed changes consistent with a widespread pneumonic process with an accompanying small pneumothorax. Lung biopsy revealed a severe distortion of the lung architecture, with features most consistent with a chronic pneumonitis of infancy. A brain MRI showed generalised atrophy with mild hyperintensity of the parieto-occipital white matter. A muscle biopsy was unremarkable, with normal mitochondrial staining and appearances. A liver biopsy showed increased glycogen in the hepatocytes and some abnormal flocculant material within the lysosomes but was otherwise normal. Molecular genetic studies were not performed.

# Case report

***Patient 6* (Family 4)** is a now 6-year-old female of nonconsanguineous parents from Slovakia. She first presented at the age of 2 months in our hospital with severe haemolytic, macrocytic anaemia (HB 65 g/l) with marked aniso- and poikilocytosis, feeding problems, failure to thrive and muscular hypotonia. The family history was remarkable in that her brother (patient 7) died from severe septic complications at the age of 10 months. Subsequently, she suffered from acute respiratory distress syndrome (ARDS) at the age of 4 months (*pneumocystis carinii* and *streptococcus pneumonia*; ICU care and ventilation). Myoclonic convulsions of the upper extremity started at the age of 7 months. Anticonvulsive therapy was started at the age of 1 year 11 months because of the highly abnormal waveforms seen in EEGs. Furthermore, microcephaly was noted during the follow-up (OFC was 35 cm/ + 1 SD at birth, and 43 cm/-3.5 SD at the age of 2.5 years). Later on, at the age of 3 years and 10 months, she developed epileptic (tonic-myoclonic) seizures. Initial haematology findings (see Figure 3) in the peripheral blood and bone marrow were suggestive of CDA type II, but no pathogenic variants were identified in CDA II–causing genes. Because the combination of CDA type II–like disease and neurological abnormalities was suggestive for CAD associated uridine responsive epileptic encephalopathy (Koch et al. 2017), uridine treatment was initiated. However, no disease-causing variants were subsequently identified in the *CAD* gene, and no response to therapy was observed.

She received dual antibiotic prophylaxis against *pneumocystis carinii* and *pneumococcus* after she suffered from PCP and pneumococcus pneumonia at the age of 4 months. Because of transient B-lymphopenia and low IgG levels, IG was substituted IV transiently. She continues to have low IgM levels.

***Laboratory investigations and imaging.*** Brain MRI at the age of 2 years revealed an arachnoidal cyst in the left temporal region and atrophy of the left temporal lobe (Figure 5). A WES analysis revealed compound heterozygote variants in the *NHLRC2* gene (paternal NM_198514.3:c.338T>G, NP_940916.2:p.Leu113Arg, heterozygote; maternal NM_198514.3:c.442G>T, NP_940916.2:p.Asp148Tyr, heterozygote).

***Current status.*** Patient 6 is currently 6 years old and lives at home with her parents. At the age of 5 years, a PEG probe was inserted because of feeding problems, and thereafter, she rapidly gained weight (4 kg in 8 months; body length 10th percentile; body weight 25th percentile). Oral cefaclor prophylaxis has been given because she suffered from a severe septic episode in infancy, and she has not had any severe infections during the past few years. Her haematological phenotype is unchanged, which is a mild macrocytic anaemia with marked anisocytosis and poikilocytosis (similar to CDAII). She suffers from severe neurodevelopment retardation and is noncommunicative, and despite broad anticonvulsive medication, she experiences three to five tonic-clonic seizures per day, which resume spontaneously. The family is supported by an outpatient palliative care team.

# Case report

**Patient 7** was the brother of patient 6. He was the first child of non-consanguine Caucasian parents. Besides delayed development and feeding problems, he suffered two times from *E.coli* meningitis/sepsis (neonatal sepsis and relapse). He had received several RBC transfusions because of severe anaemia with marked anisocytosis and poikilocytosis. At the age of 10 months, he presented with fever and enteritis and subsequently died from sepsis with multiorgan failure.

***Laboratory investigations and imaging.*** Post-mortem Sanger sequencing on a histopathological autopsy specimen revealed that he carried the same compound heterozygous *NHLRC2* variants (paternal NM_198514.3:c.338T>G, NP_940916.2:p.Leu113Arg, heterozygote; maternal NM_198514.3:c.442G>T, NP_940916.2:p.Asp148Tyr, heterozygote) as his sister (patient 6). Reports from the blood smears describe the same picture as his sister (patient 6): marked anisocytosis and poikilocytosis with the presence of tear drop cells and target cells.

# Case report

***Patient 8*** (Family 5) is the 61-year-old male who was re-examined because of previously undefined profound mental retardation with autistic features, spastic diplegia, epilepsy and chronic anaemia. The etiological investigations revealed the homozygous variant (c.442 G>T, p.D148Y) in the *NHLRC2* gene. Thus, he is the oldest FINCA patient who has been found so far.

He is the first (1/III) child of healthy unrelated Finnish parents. The patient was born at 42+2 weeks in the breech position after prolonged delivery. The Apgar scores were not available, but he was cyanotic, initially resuscitated and needed oxygen supply, and breastfeeding was not successful during the first two days. Birth weight was 3570 g, birth height 50 cm and head circumference 36 cm. Before discharge, the paediatrician did not notice any abnormalities. He was able to support his head by the age of 2 months, and at the age of 8 months, he began to crawl. He learned to walk at the age of 17 months, but he had balance problems. At the age of 10 months, the patient began to have occasional convulsive seizures, and phenobarbital-phenytoin combination was started as an anticonvulsant drug. The initiated medication had a good response, and the seizures temporarily disappeared. Developmental problems were not detected at the time. By the age of 3 years, the patient had not learned to speak. Since then, the seizures reappeared, and at the age of 4, he was diagnosed with epilepsy with generalised tonic-clonic seizures, and his development was found to be at the level of 1.5 years. Furthermore, lower limb spasticity was noted. At the age of 7, the patient was diagnosed with a profound intellectual disability. He underwent chromosomal and metabolic investigations, but these did not reveal any explanation. The cause of severe intellectual disability was considered to be prolonged delivery and birth asphyxia. His growth was regular and stayed within the lower percentiles.

Because of remarkable behavioural problems since early childhood, the patient has been in institutional care since the age of 7. He first began to behave aggressively towards his younger siblings, and since then, he has been reserved and unpredictable in the company of children and adolescents. From the age of 10 onwards, he has suffered from frequent sudden anger bursts, in which case he is violent and may bite. He understands simple promptings, expresses himself with gestures and mimics sounds. The only meaningful word used is ‘dad’, and he eats untidily but independently, seeks affection and attention from adults.

In childhood, the patient had a tendency to get various bacterial infections, and in adolescence, at the ages of 9–16, he had several subcutaneous infections, including subcutaneous abscesses, recurrent pharyngitis and peritonsillar abscesses and gastroenteritis. He underwent tonsillectomy at the age of 11 and appendicectomy at the age of 21. During adulthood, he has had several pneumonias and other severe infections (CRP elevation up to 250 mg/l) with undefined focus and aetiology, and clinical examination has been very challenging because of difficult cooperation with the patient. The most recent pneumonias have been during the past few years, and on both occasions, an intravenous antibiotic treatment was needed. Furthermore, he has a history of recurrent urinary tract infections without any specific risk factors (ultrasonography showed normal kidneys, bladder and prostate), skin rash problems and poor teeth condition, which led to the removal of his teeth. He has also had frequent problems with stomach and bowel function, and during the past few years, he has had ileal occlusion. Because of behavioural problems, sudden aggressive bursts, epileptic seizures, spasticity and balance problems, he has had several accidents resulting in variable contusions and a fracture of his right femur. Focal onset, secondarily generalised tonic-clonic seizures (GTC) became more frequent at the age of 4 and continued until the age of 10, after which the patient did not present with GTC seizures for several decades, but instead, aggressive outbursts became the predominant feature of the disease, occurring weekly on average. These episodes last less than half an hour, after which the patient is often laughing and feeling relieved. The electroencephalogram (EEG) was performed for the first time at the age of 4, and the following findings have been constant during the follow-up: multifocal epileptic activity, irritative bursts and sharp waves, especially in frontal and occipital regions with frontal dysrhythmia. Initially, at the age of 10 months, a combination of phenobarbital (PB) and phenytoin (PHT) was started as an antiepileptic drug. At the age of 14 years, PB/PTH treatment was replaced by carbamazepine (CBZ). During CBZ treatment, the patient did not have any GTC seizures, but the treatment response was not achieved for aggressive bursts. At the age of 52 years, the patient started to present with absence seizures, followed by reoccurrence of GTC seizures. Therefore, CBZ was replaced by lamotrigine (LTG). The rage outbursts continued, but after the initiation of LTG, they were not as frequent as before. Benzodiazepines, including midazolam, have had a good response to GTCs without progression to status epilepticus.

***Laboratory investigations and imaging.*** At the age of 40 years, he presented with decreased blood haemoglobin (124 g/l, reference 134–167 g/l) without any signs of infection (normal values in blood leucocytes and in C-reactive protein test). Initially, iron supplementation for anaemia was started, but haemoglobin remained at the levels of 120–130 g/l, and chronic megaloblastic anaemia was noted in the follow-up investigations. In the latest blood test at the age of 60 years, the patient continued to have megaloblastic, normochromic anaemia. At the age of 11, pneumoencephalography was performed, and the findings were within the normal range.

***Current status.*** The patient is still alive at the age of 61. During the years of institutionalisation, there have been no significant changes in his behaviour or skills, but some progression has been noted in upper motor neuron functions and balance. The current medication includes lamotrigine 100 mg x 3 for epilepsy, olanzapine 10 mg x 3 as a sedative and sertraline 25 mg every other day as an antidepressant. He also has midazolam and temazepam to be taken, if needed (for seizures and agitation, respectively).

At the last follow-up clinical examination (at the age of 61 years), his height was 169 cm, he had no obesity, his facial appearance was hypomimic, his mouth stayed open and generalised facial muscle hypotonia was noted. He sits calmly and holds a sock in his hand for safety. He had fallen the day before because of a GTC seizure, so he had haematoma and swelling around his left eye. He recognises his own name and occasionally reacts to speech with gestures, hand clapping and sounds. He has neuromuscular scoliosis, remarkable pectus excavatum and generalised muscular atrophy that is prominent, especially in the proximal limbs, in the shoulders and in the back. In his movements, right-sided prominence is noted, and the left side is more spastic. Hyperreflexia is noted in lower limb deep tendon reflexes, and the left patellar reflex is brisker than the right. Babinski’s sign is positive on the left. Furthermore, he has stereotypical midline hand manoeuvres. His gait is wide and atactic, and he walks with his hips and knees flexed and his feet turned inwards. His eye movements are normal, and he does not have strabismus. Cardiac and pulmonary auscultations were normal.
