# Supplementary figures and images for: Novel patients with NHLRC2 variants expand the phenotypic spectrum of FINCA disease
Source: Front Neurosci. 2023 Apr 27;17:1123327. doi: 10.3389/fnins.2023.1123327 (PMC10173879; doi:10.3389/fnins.2023.1123327)

Supplementary figure 1

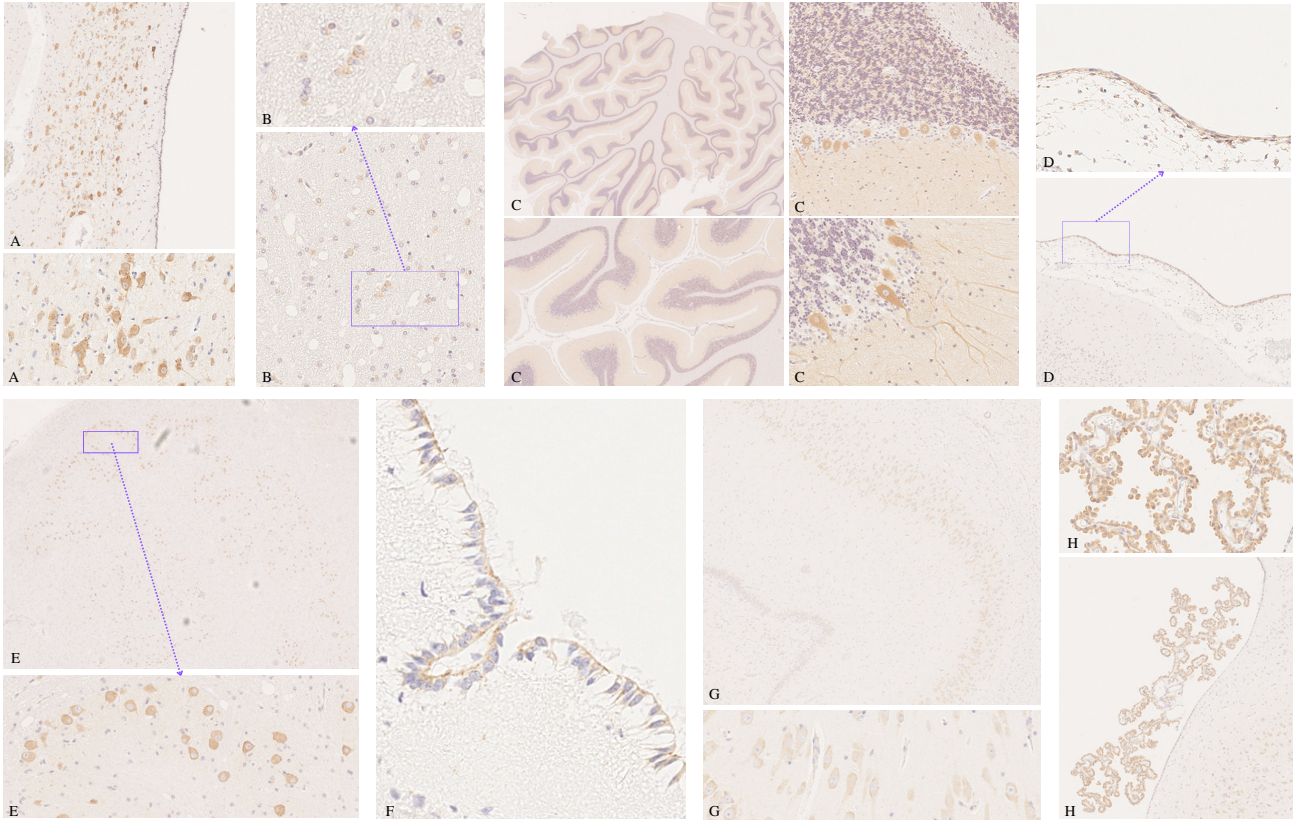

Supplementary figure 2

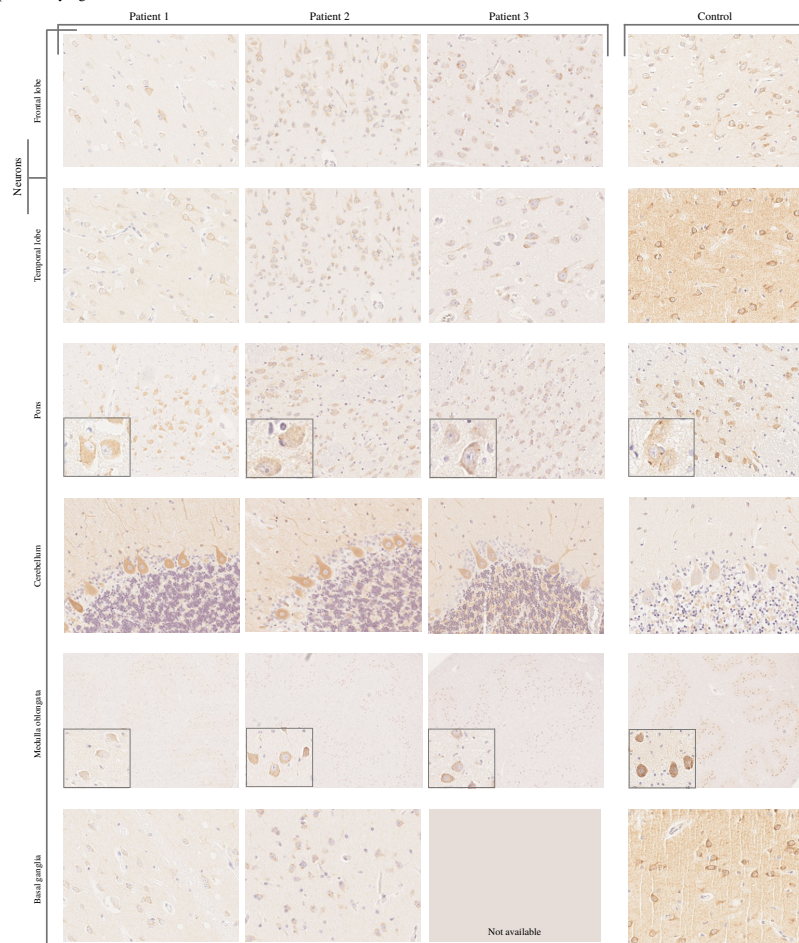

Supplementary figure 3

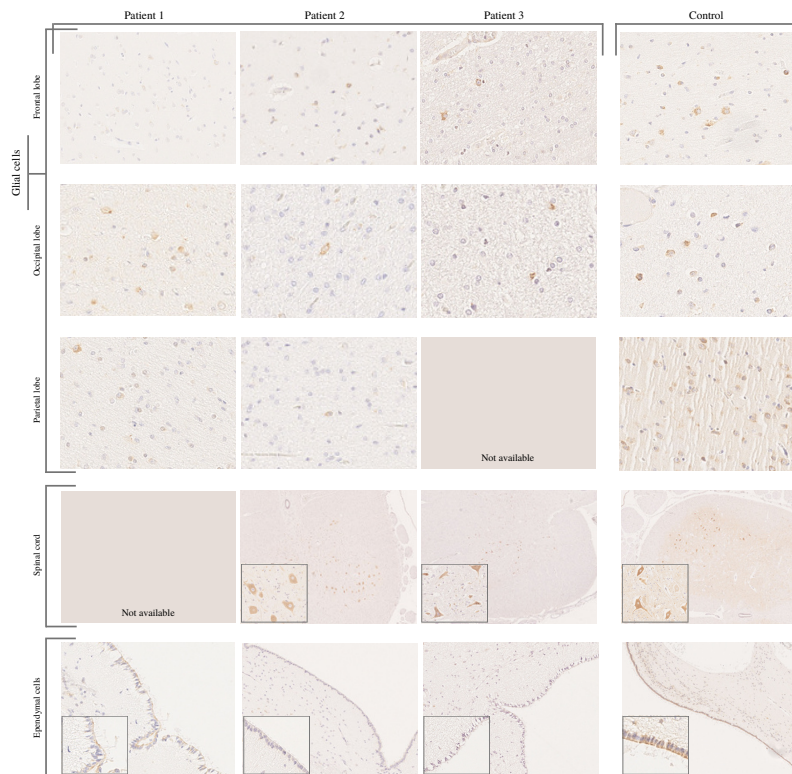

Supplement: Supplementary Figure 1 — Immunohistochemical NHLRC2 expression in brain autopsy samples of patients. (A) Strong expression in thalamic neurons. (B) Medium strength and widespread expression in the glial cells of the middle brain. Some glial cells are weakly positive or negative for NHLRC2. (C) Cerebellar overview and higher magnifications of the cell layers and Purkinje cells. Strong expression in Purkinje cells. (D) Meningothelial cells are weakly positive for NHLRC2. (E) Neurons of the inferior olivary nucleus have medium to strong expression. (F) Ependymal cells had apical expression and were weakly positive or negative for NHLRC2. (G) Dentate gyrus of the hippocampus and mild expression in neurons. (H) Expression in choroid plexus cells and periventricular neurons. [file Image_1.pdf]
